# Dynamising One Health Through a Structured Global Networking Model

**DOI:** 10.3390/vetsci13070720

**Published:** 2026-07-22

**Authors:** Ulrich Laaser, Nedjeljko Karabasil, Helmut Wenzel, Vesna Bjegović Mikanović

**Affiliations:** 1 Faculty of Health Sciences, Bielefeld University, D-33501 Bielefeld, Germany; ulrich.laaser@uni-bielefeld.de; 2 Faculty of Veterinary Medicine, University of Belgrade, 11000 Belgrade, Serbia; 3 Independent Consultant, 78462 Konstanz, Germany; hkwen@aol.com; 4 Faculty of Medicine, University of Belgrade, 11000 Belgrade, Serbia; vesna.bjegovic-mikanovic@med.bg.ac.rs

**Keywords:** One Health governance, community health, veterinary public health, environmental health, global health networks, implementation science

## Abstract

Global environmental change, biodiversity loss, emerging infectious diseases, antimicrobial resistance, and widening social inequalities increasingly interact to create complex risks for human, animal, and ecosystem health. Although international One Health frameworks and funding mechanisms are essential, implementation at national and especially community levels often remains uneven, fragmented, and weakly evaluated. This Perspective presents a structured, reproducible networking model designed to strengthen community-level One Health implementation through voluntary global collaboration among civil society organisations. The model connects bottom-up community mobilisation with interdisciplinary scientific support, shared evaluation templates, and iterative learning cycles. It outlines a transparent process for identifying and engaging One Health-oriented organisations and for organising their collaboration through thematic Project Working Groups that support situation analysis, strategic planning, implementation, evaluation, and dissemination. The framework translates community action into seven intervention fields: surveillance and early warning, animal health and welfare, water/sanitation/hygiene, food safety and food systems, community awareness and education, environmental risks and ecosystem health, and multisectoral governance. By linking local practice to comparative synthesis, joint publication, and policy dialogue, the network aims to transform isolated initiatives into a coordinated learning system while preserving community and organisational autonomy. As a conceptual framework, the model is ready for empirical testing in diverse settings to assess feasibility, participation, and evaluation quality.

## Simple Summary

Environmental change, biodiversity loss, emerging infectious diseases, antibiotic resistance, and growing inequalities are increasingly interconnected, creating growing risks for people, animals, and ecosystems. Global One Health strategies and funding mechanisms are important, but implementation at the local community level remains uneven, poorly coordinated, and weakly evaluated. We propose a practical, repeatable model that strengthens community-level One Health action through voluntary global collaboration among civil society organisations. The model links local mobilisation with interdisciplinary scientific support, shared monitoring and evaluation templates, and iterative learning cycles, organised through themed Working Groups. The framework focuses on seven goal-based areas: surveillance and early warning; animal health and welfare; water, sanitation and hygiene; food safety and food systems; community awareness and education; environmental risks and ecosystem health; and multisector governance. By connecting local projects with shared analysis, joint publications, and policy dialogue, the network can transform initiatives into a coordinated learning system while preserving community and organisational independence.

## 1. Introduction

Human-driven degradation of water, soils, biodiversity, and the atmosphere is reshaping living conditions worldwide, with rising temperatures, ecosystem disruption, and pollution affecting both health outcomes and social stability [1]. Plastics and microplastics have now been detected across ecosystems, food chains, and human biological samples, adding further dimensions to environmental and health risk [2,3]. Over the past two decades, it has become evident that many of these interacting trends may reach irreversible tipping points before political consensus and large-scale intervention can be achieved. Although the Sustainable Development Goals (SDGs), adopted in 2015, provide an overarching global framework, progress toward many targets remains insufficient and uneven [4]. Gains in selected areas such as education and digital access coexist with persistent poverty, food insecurity, inadequate housing, and unequal access to basic services, disproportionately affecting women, marginalised populations, and low-resource communities. Climate impacts, armed conflicts, and high public debt servicing costs further constrain national and regional capacities for coordinated response.

The COVID-19 pandemic illustrated how rapid local disruptions can cascade into global crises, while also revealing structural weaknesses in preparedness, governance, and cross-sectoral coordination. In response, One Health has gained prominence as an integrated framework linking human, animal, plant, and environmental health. The Quadripartite (FAO, UNEP, WHO, and WOAH) defines One Health as an approach that aims to sustainably balance and optimise the health of people, animals, and ecosystems by recognising their interconnectedness and interdependence [5]. While this definition is widely endorsed, implementation has often remained narrow in scope, frequently centred on infectious disease preparedness and surveillance, with limited integration of social, environmental, and governance determinants. At the same time, many One Health initiatives remain localised, time-limited, and insufficiently evaluated. Reviews of One Health implementation studies have highlighted a lack of micro-level analyses, limited methodological transparency, and weak comparability across settings [6]. As a result, valuable local experience is rarely aggregated into transferable knowledge, scalable models, or policy-relevant guidance. This fragmentation is particularly evident at the community level, where households, schools, municipalities, civil society organisations, and local service providers often act faster than higher governance tiers but lack structured scientific support, evaluation frameworks, and channels for systematic exchange [7,8]. Civil society organisations (CSOs) and non-governmental organisations (NGOs) occupy a critical intermediary position between communities, science, and policy. Globally, the One Health movement comprises a large and diverse set of CSOs/NGOs with complementary mandates and expertise. However, cooperation among these actors remains largely informal and uncoordinated. Limited visibility, duplication of effort, and weak linkage to both community practice and international governance reduce collective effectiveness [9,10].

This paper addresses these gaps by presenting a structured, reproducible networking and coordination model that integrates community-based One Health action with voluntary global collaboration among CSOs/NGOs. Rather than proposing a new institutional hierarchy, the model focuses on enabling exchange, harmonisation, and collective learning across existing actors. The objective is to operationalise One Health at the community level while simultaneously strengthening coordination, evaluation, and dissemination through a global civil society network. The framework is designed to be adaptable across diverse contexts and to provide a methodologically sound basis for future empirical testing and evaluation.

## 2. Conceptual Framework

The proposed approach is a dual-level networking model that links (i) community-based One Health action and research with (ii) voluntary global coordination among One Health-oriented CSOs/NGOs. The model is conceptual and methodological in nature, describing a structured process that can be replicated, adapted, and empirically tested in different geographical and socio-political contexts. No primary human or animal data were collected for this study.

The framework is guided by three principles: (i) Bottom-up empowerment, whereby communities co-define priorities, interventions, and evaluation needs; (ii) Voluntary coordination, preserving organisational autonomy while enabling structured exchange and cooperation; (iii) Continuous learning, integrating implementation, evaluation, and improvement cycles.

The scientific rationale for this framework is grounded in implementation science, public health governance, and transdisciplinary One Health research. From an implementation science perspective, the central challenge is not only to define One Health priorities, but to also create mechanisms through which evidence can be adapted, applied, evaluated, and improved in real-world settings. The use of community action cycles, SMART-based planning, and iterative evaluation reflects this logic by linking local problem definition with measurable objectives, feedback, and continuous learning. From a public health governance perspective, non-hierarchical and facilitative coordination is appropriate for complex health risks that cross institutional, sectoral, and geographical boundaries. Such risks cannot be effectively addressed through single-sector or top-down structures alone, because they require trust, shared interpretation, and coordinated action among actors with different mandates and forms of knowledge. Finally, transdisciplinary One Health and Ecohealth approaches support the use of Project Working Groups as boundary-spanning structures that connect community experience, scientific expertise, policy dialogue, and practical implementation [11,12]. In this sense, the proposed organisational mechanics are not presented as administrative procedures, but as functional mechanisms for translating integrated knowledge into locally relevant and evaluable One Health action.

### 2.1. Community-Level Action Cycle

Community-level action cycles are included because effective One Health implementation depends on the capacity of local actors to identify risks, prioritise feasible responses, test interventions, and adapt them over time. This reflects implementation science principles, in which interventions are not treated as fixed packages, but as processes that must be adapted to real-world conditions, resource constraints, institutional arrangements, and local social and ecological contexts. In Ecohealth and transdisciplinary One Health research, communities are not only recipients of expert recommendations; they are also knowledge holders who can identify environmental changes, animal health concerns, behavioural practices, service gaps, and early warning signals that may not be visible through formal surveillance systems alone [11,12].

At the community level, the model operationalises One Health through a cyclical process consisting of three interconnected phases. First, during mobilisation and situation analysis—local administrations, CSOs, public health and veterinary services, environmental management, educators, and community representatives—conduct a structured situation analysis. This includes health, environmental, and social determinants relevant to One Health, with attention to inequalities and vulnerable groups. Second, based on the assessment, communities select one or more intervention fields from a predefined but adaptable menu. Interventions are framed using SMART principles (Specific, Measurable, Achievable, Relevant, Time-bound) to facilitate planning and evaluation. Third, implementation is accompanied by proportionate mixed-methods evaluation allowing communities to document what was done, what changed, what barriers were encountered, and how interventions should be refined. The SMART-based planning matrix is used as a pragmatic implementation science instrument. It helps communities translate broad One Health priorities into concrete, measurable, achievable, relevant, and time-bound actions. This is particularly important in heterogeneous and low-resource settings, where evaluation must remain feasible while still producing information that can support learning and comparison across sites. By linking objectives, indicators, available resources, responsibilities, and timelines, the matrix connects local planning with accountability and continuous improvement. Findings are used to refine interventions locally and are documented for comparative analysis and dissemination within the network [13]. In this way, the community-level action cycle provides a scientific rationale for locally driven, adaptive implementation rather than one-directional, centrally prescribed action.

### 2.2. Global Coordination of CSOs/NGOs

Zinsstag et al. [14] emphasise that One Health problems emerge from complex social–ecological systems and therefore require transdisciplinary approaches that integrate community-based knowledge with scientific and policy expertise; this directly supports our model in which CSOs, and NGOs collaborate with interdisciplinary groups to generate context-appropriate and operationally feasible solutions.

To support and connect community-level action, a voluntary global coordination mechanism among One Health-oriented CSOs/NGOs is proposed. The coordination process is designed as a transparent, stepwise method that can be reproduced by other groups or regions.

### 2.3. Identification and Engagement of Organisations

Potential participating organisations are identified through publicly available global databases, including One Health-focused organisational listings and the UN ECOSOC (United Nations Economic and Social Council) civil society registry. Organisations are screened using predefined criteria, including One Health orientation, independence from direct control by governments and commercial entities, transparency about funding sources, engagement beyond single diseases or species, and multi-country or global scope. Detailed criteria and screening procedures are provided in Appendix A.1. A transparent engagement procedure is used to invite eligible organisations to participate voluntarily. Operational details, including the invitation template and replacement procedure for non-response, are provided in Appendix A.

### 2.4. Information Sharing and Transparency

Participating organisations would contribute a concise description of their mandate, governance arrangements, areas of activity, cooperation partners, and recent publications and outputs. This shared information is intended to improve mutual visibility and trust, and to help organisations identify opportunities for facilitation of cooperation and collaboration. It is not intended as a process of accreditation, ranking, or formal evaluation. A possible information template and invitation letter are provided in Appendix A.2.

### 2.5. Project Working Groups (PWGs)

Project Working Groups (PWGs) are included as boundary-spanning and translational structures within the One Health network. Their role is grounded in transdisciplinary research, where complex problems are addressed through structured interaction among different sectors, disciplines, and forms of knowledge. One Health challenges rarely fit within the mandate of a single institution or profession. They require the integration of public health, veterinary medicine, environmental sciences, social sciences, education, policy, and community experience. For this reason, PWGs are not conceived as administrative committees, but as functional spaces where fragmented evidence can be compared, interpreted, and translated into feasible local action. The scientific value of PWGs lies in their capacity to connect knowledge generation with implementation. They provide a mechanism through which local observations, professional expertise, scientific evidence, and policy needs can be brought into dialogue. This is particularly important for One Health because relevant information often emerges from different sources, including community experience, animal health services, environmental monitoring, public health surveillance, educational institutions, and civil society organisations. By bringing these sources together, PWGs can support joint interpretation of risks, identification of implementation barriers, development of shared tools, and translation of findings into prevention, preparedness, education, and policy-oriented outputs.

PWGs function as time-bound, task-oriented units that support both community-level action and cross-organisational learning. Proposed PWGs include: PWG1—Community engagement and situation analysis (Exchange and synthesis of community assessments; stakeholder engagement methods); PWG2—Strategic planning and policy support (Development of shared planning frameworks; linkage between community action and policy dialogue); PWG3—Evidence synthesis and evaluation methods (Literature review; development and refinement of evaluation approaches; comparative analysis); PWG4—Implementation and improvement (Analysis of implementation processes; feedback of evaluation results into practice); PWG5—Curriculum development and educational resources (Development of modular One Health curricula and repositories for training institutions); PWG6—Publications, communication, and global networking (Joint scientific outputs, public communication, and alliance-building toward a Global One Health Alliance). PWGs meet predominantly online, with periodic coordination meetings to consolidate progress.

### 2.6. Governance, Coordination Mechanisms and Community Intervention Fields

The governance design is intentionally non-hierarchical, voluntary, and locally anchored. This reflects the nature of One Health problems as complex, adaptive, and context-dependent challenges that cross human, animal, environmental, institutional, and geographical boundaries. From a public health governance perspective, such problems require coordination models based on trust, shared interpretation, distributed responsibility, and local adaptation. A centralised structure may improve administrative control, but it can also reduce flexibility, weaken local ownership, and overlook context-specific risks. In contrast, facilitative governance allows local actors to retain decision-making authority while benefiting from shared tools, peer learning, technical support, and policy alignment.

Governance within this framework is therefore based on facilitation rather than control. The aim is not to govern communities from above, but to create conditions in which different actors can coordinate effectively without losing autonomy. Central or higher-level structures, where present, should provide guidance, technical support, methodological tools, and policy coherence, while practical decision-making, ownership, resource mobilisation, and implementation remain primarily anchored at the local or regional level. This arrangement is consistent with the bottom-up logic of One Health implementation and helps ensure that interventions remain responsive to local risks, capacities, and priorities. Coordination mechanisms are deliberately light and flexible. They may include regular online coordination meetings, an Organising Committee with rotating membership, and, where feasible, a small coordinating secretariat to support communication, documentation, and continuity. These mechanisms are intended to maintain transparency, enable peer learning, and support collaboration across Project Working Groups without creating a permanent central authority. Decisions should preferably be made by consensus; where full agreement is not possible, parallel approaches may be allowed, reflecting the voluntary and adaptive nature of the network. A more detailed description of the proposed governance architecture, coordination cycles, and collaborative arrangements is provided in Appendix A.3.

Community intervention fields provide the practical entry points through which local actors translate One Health principles into action. The initial menu of seven areas covers surveillance and early warning, animal health and welfare, water and sanitation, food safety and food systems, community awareness and education, environmental risks and ecosystem health, and multisectoral coordination. These fields are intended as a flexible menu rather than a fixed prescription. Communities may select, combine, adapt, or expand them according to their own epidemiological, ecological, social, and institutional contexts. Each field can be framed using SMART criteria to ensure that local actions are feasible, evaluable, and comparable where appropriate. This combination of local choice and shared methodological structure supports both contextual adaptation and cross-site learning. An illustrative intervention matrix is included in Appendix A.4 to show how these fields may be translated into locally adaptable planning and evaluation tools.

The One Health Joint Plan of Action (2022–2026) issued by the Quadripartite, defines six global Action Tracks. Our seven community intervention fields are broadly aligned with these institutional priorities, demonstrating how the CSO model operationalises the same governance, coordination and risk-reduction domains endorsed at the international policy level [15]. Global structures in light of One Health networks remain uneven, hierarchical, and poorly aligned with local needs. Most networks are concentrated in high-income regions, prioritise emerging infections over food safety and environmental degradation, and rarely include community-level actors, which significantly weakens the effectiveness of One Health interventions [10]. One Health challenges arise within complex social–ecological systems where risks are shaped by local environmental conditions, community practices, and socio-economic dynamics. Because such systemic interactions cannot be understood or managed through top-down sectoral structures, the authors argue for governance models that integrate local knowledge with interdisciplinary expertise [16]. The operationalisation of One Health is consistently hindered by rigid institutional silos, fragmented mandates, and the absence of mechanisms that enable genuine cross-sectoral collaboration. Without structures capable of integrating local knowledge, environmental signals, and community-level practices, One Health remains largely theoretical and fails to translate into effective action [17]. Globally, the One Health concept and Planetary Health have expanded rapidly but remain uneven, dominated by high-income academic institutions and constrained by narrow thematic focus, with mostly zoonoses on one side and climate-related determinants on the other. Crucially, both fields lack strong operational links to communities and non-academic actors, which limits their ability to translate systemic insights into practical action [18].

Our bottom-up CSO approach directly reflects this logic: by positioning community actors as primary observers of ecosystem change and co-designers of interventions, the model operationalises the systemic, context-responsive governance principles. In this way, the Project Working Groups (PWGs) become the institutional mechanism through which local insights and scientific capacities are merged into coherent, actionable One Health strategies that connect sectors where risks actually emerge and decisions must be implemented.

## 3. Proposed Model

Building on these implementation science, governance, and transdisciplinary principles, the model is presented as a testable framework for organising, comparing, and improving community-level One Health action across diverse settings.

The proposed model results in a multi-layered One Health coordination architecture that connects community-based action with global civil society collaboration. The architecture is intentionally designed as a distributed network, rather than a centralised organisation, allowing participation by heterogeneous actors while preserving autonomy. At the community level, local action groups implement One Health interventions adapted to local priorities and capacities. These groups interact horizontally with peer communities through shared tools, evaluation frameworks, and thematic exchanges. At the global level, participating CSOs/NGOs form a voluntary coordination forum that provides facilitation, methodological support, and aggregation of experience. The two levels are linked through Project Working Groups (PWGs), which function as operational bridges between local practice and global learning.

The model generates a structured flow of information across levels: (i) Communities conduct situation analyses and select intervention fields; (ii) Implementation and evaluation data are documented using shared templates; (iii) PWGs synthesise findings across communities and organisations; (iv) Results are fed back to communities as improved guidance, tools, and curricula; (v) Aggregated insights inform joint publications, policy dialogue, and global advocacy. This feedback structure transforms isolated projects into a coordinated learning system without imposing uniform interventions or indicators.

### 3.1. Functioning of Project Working Groups

**Figure 1:**
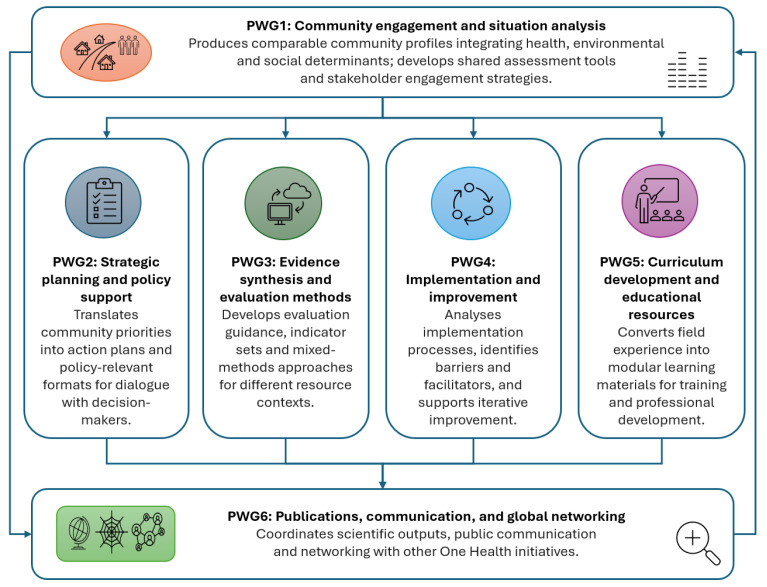
Links of exchange and cooperation between the six working groups are proposed. Each PWG has a clearly defined mandate, expected outputs, and time horizon. Arrows show the direction of collaboration: PWG1 provides community input and strategic direction to the four thematic groups (PWG2–PWG5), whose outputs feed into PWG6 for publication and global networking. The diagram also highlights continuous cross-collaboration among all PWGs, including a direct link between PWG1 and PWG6, ensuring that community insights, thematic outputs, and global communication inform one another throughout the process.

PWGs are the core operational units of the network (Figure 1). In this framework, PWGs function as translational and learning units rather than administrative committees. Their purpose is to connect evidence generation, field experience, evaluation, and policy communication in a way that allows knowledge to move in both directions: from science to practice and from community practice back to research and policy. Each PWG has a clearly defined mandate, expected outputs, and time horizon. Membership is open to participating CSOs/NGOs and affiliated experts, allowing interdisciplinary collaboration.

PWG1 (Community engagement and situation analysis) produces comparable community profiles that integrate health, environmental, and social determinants. Outputs include shared assessment tools and stakeholder engagement strategies. PWG2 (Strategic planning and policy support) translates community priorities into structured action plans and policy-relevant formats, facilitating dialogue with local, national, and international decision-makers. PWG3 (Evidence synthesis and evaluation methods) develops and updates evaluation guidance, including indicator sets and mixed methods approaches appropriate for varying resource contexts. PWG4 (Implementation and improvement) analyses implementation processes, identifies barriers and facilitators, and supports iterative refinement of interventions. PWG5 (Curriculum development and educational resources) converts field experience into modular learning materials for training institutions and professional development. PWG6 (Publications, communication, and global networking) coordinates scientific outputs, public-facing communication, and alliance-building with other One Health initiatives. PWGs operate predominantly through online collaboration, enabling global participation with limited resource requirements.

Communities engage with the network by selecting one or more intervention fields as entry points. The seven proposed fields provide a menu rather than a prescription, enabling contextual adaptation. Each field is structured to allow: (i) Clear definition of objectives and target populations; (ii) Selection of feasible indicators; (iii) Documentation of implementation processes; (iv) Comparison across settings where appropriate. The use of SMART principles supports pragmatic planning and evaluation while avoiding excessive standardisation. Communities may expand or modify intervention fields as priorities evolve.

The proposed governance structure results in several functional outcomes: (i) Transparency (A shared minimum dataset on participating organisations improves visibility and trust); (ii) Continuity (Regular coordination cycles and PWGs sustain engagement beyond short-term projects); (iii) Scalability (The network can expand gradually by adding communities, organisations, or PWGs without structural redesign); (iv) Inclusiveness (Voluntary participation and online collaboration lower barriers for low-resource settings and underrepresented groups). Rather than creating a new institutional actor, the model strengthens the connective tissue among existing initiatives.

### 3.2. Anticipated Outputs and Readiness for Empirical Testing

Until it is tested in real communities, organisations, and governance settings, the model should be regarded as a hypothetical and testable framework rather than a validated intervention. As this manuscript presents a Perspective and conceptual framework rather than empirical pilot data, the outputs described below should be understood as anticipated products of future implementation, not as demonstrated results. Their purpose is to define what the model is expected to generate if applied and evaluated in real-world settings, and to identify suitable domains for future empirical testing. In this sense, the anticipated outputs are not claims of effectiveness, but proposed indicators of feasibility, learning, coordination, and translational value. An illustrative AMR pilot scenario is provided in Appendix A.4 to show how the SMART matrix could be translated into concrete objectives, data fields, indicators, anticipated outputs, and evaluation domains in a One Health problem relevant to human, environmental, and veterinary health.

At the community level, the model is expected to generate documented interventions, evaluated summaries, and locally relevant improvements.

At the network level, it is expected to support comparative analyses, shared tools, and harmonised evaluation approaches.

At the global level it may contribute to joint scientific publications, educational resources, coordinated policy-relevant messages, and greater visibility of community-based One Health experience.

**Figure 2:**
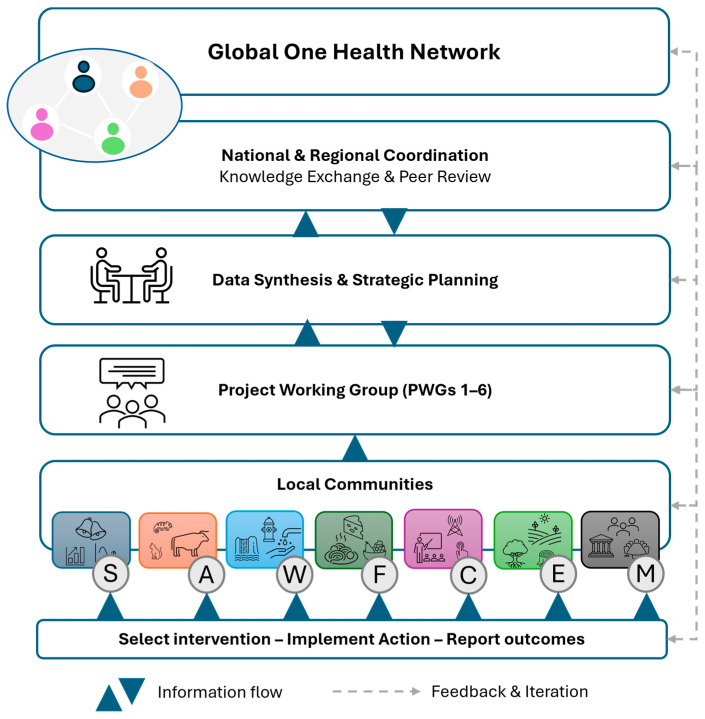
Conceptual model of the global One Health network. The figure illustrates the multi-layered network architecture linking community-level action with global CSO/NGO coordination. Communities select intervention fields, implement actions, and report outcomes via shared templates. The seven community intervention fields are abbreviated as S-A-W-F-C-E-M: S, Surveillance and early warning; A, Animal health and welfare; W, Water, sanitation, and hygiene; F, Food safety and food system; C, Community awareness and education; E, Environmental risks and ecosystem health; M, Multisectoral coordination and governance. Project Working Groups (PWGs) synthesise data, support strategic planning, and provide feedback and improvements. The network facilitates knowledge exchange, peer learning, and dissemination of best practices while preserving community autonomy. Solid arrows indicate information flow; dashed arrows indicate feedback and iterative improvement cycles.

These outputs are intended to be openly accessible, supporting transparency and reuse. The conceptual model of the global One Health network is shown in Figure 2. The model’s components—community action cycles, CSO/NGO coordination procedures, PWGs, and governance mechanisms—are sufficiently specified to allow empirical testing. Implementation studies may assess feasibility, participation patterns, quality of evaluation, and added value compared with uncoordinated One Health initiatives.

This collaborative structure also has clear translational research value, because it helps turn evidence from different sectors into practical prevention, preparedness, and response measures at the local level. Rather than treating information collection as an end in itself, the network brings together community observations, veterinary and public health surveillance, environmental monitoring, laboratory findings, and scientific expertise to support evidence-based interpretation and prediction. In outbreak investigation, for example, locally reported signals from communities, animal health services, environmental actors, and public health institutions can be analysed together to identify possible sources of exposure, transmission pathways, vulnerable settings, and early warning patterns. In antimicrobial resistance, the same structure can connect data from human health care, livestock production, food systems, wastewater, wildlife, and environmental samples to support risk mapping and targeted interventions. For vector-borne and transboundary diseases, it can help compare local observations with regional trends in climate, land use, animal movement, trade, and vector distribution. Similarly, information on biodiversity loss, habitat change, pollution, and ecosystem degradation can be translated into locally relevant action for ecosystem protection and resilience. In this way, the network creates a practical pathway from knowledge generation to coordinated local implementation, while also allowing field experience to shape future research priorities and policy dialogue.

## 4. Relevance to Global Health and Sustainability

A structured model is needed for a global One Health network linking community-based initiatives with civil society organisations (CSOs/NGOs) and international governance frameworks. The model addresses persistent gaps in One Health implementation: fragmentation, limited cross-sectoral learning, and underutilisation of local knowledge. By formalising information flows, Project Working Groups (PWGs), and iterative feedback loops, the network transforms isolated initiatives as mapped by the One Health Commission [19] into a coordinated learning system. A key contribution is the operationalisation of bottom-up empowerment, allowing communities to define intervention priorities while simultaneously enabling global coordination and dissemination of best practices. The model respects the autonomy of local actors while ensuring that outputs (e.g., evaluation results, curricula, joint publications) are shared to benefit wider One Health efforts.

The model aligns with priorities identified by the Quadripartite One Health framework [5] and complements ongoing UN initiatives, including the Pandemic Prevention, Preparedness, and Response Agreement [7]. It provides actionable mechanisms for: (i) Accelerating translation of research into practice; (ii) Enhancing cross-community comparability of interventions; (iii) Supporting inclusion of underrepresented communities and regions; (iv) Integrating health, environmental, and social determinants. The focus on community-level engagement reflects evidence that many global health challenges, including emerging infectious diseases, antimicrobial resistance, and environmental degradation, require locally adaptive responses, rather than top-down mandates alone.

### Advantages, Limitations and Challenges of the Model

The advantages of the proposed model are numerous: (i) Scalability—Communities and organisations can join progressively without restructuring the network; (ii) Inclusivity—Online PWG collaboration lowers participation barriers for low-resource or remote settings; (iii) Evaluation integration—Standardised templates and iterative improvement cycles allow continuous learning; (iv) Multisectoral linkage: Integration of health, veterinary, environmental, and social sciences enables interventions that reflect complex determinants of health.

The proposed network architecture should be understood not only as an organisational framework for coordination among One Health actors, but also as a practical mechanism for connecting biodiversity protection, animal health, public health outcomes, and response preparedness. By linking local, regional, and transboundary actors, the network enables environmental, veterinary, and public health information to be interpreted jointly rather than in isolation. This is particularly important because changes in ecosystems, land use, wildlife habitats, livestock systems, and vector distribution can directly influence the emergence and spread of health risks. Vector-borne and transboundary diseases provide a clear example of this interdependence, such as mosquito species, ticks, wildlife reservoirs, domestic animals, and the pathogens they carry may expand into new areas under the influence of climate change, environmental modification, animal movement, trade, and human mobility. A structured One Health network can therefore strengthen preparedness by connecting community observations, veterinary and public health surveillance, biodiversity and environmental monitoring, and local response capacities. Such integration supports earlier recognition of ecological and epidemiological signals, improves risk communication, and enables more targeted prevention and coordinated response measures across sectors and regions.

Several limitations should be acknowledged. Although the model is theoretically grounded and informed by prior One Health experience, its feasibility, effectiveness, and long-term sustainability remain to be assessed through empirical validation in real-world settings. Resource constraints may also influence implementation, as even low-cost online coordination and PWG facilitation require staff time, continuity and at least minimal financial support. In addition, heterogeneity across regulatory, cultural, and infrastructural settings may affect the extent to which the model can be adopted and implemented. Finally, maintaining consistent documentation across diverse communities may be challenging, and could influence the comparability of findings. These limitations highlight the need for iterative refinement and flexible adaptation at local and regional levels.

This model offers a practical framework for scaling bottom-up One Health initiatives, and for linking local action with global collaboration. Its further development will require empirical assessment in selected community settings, with attention to feasibility, uptake, and early implementation outcomes. Future research should also examine how digital platforms may support data sharing, communication, training and cross-site learning, as well as how PWGs contribute to synthesis and dissemination of practice-based evidence. In parallel, the model may inform broader discussions on international One Health by demonstrating how community participation, evidence generation, and global network, can be integrated into a coherent implementation framework. By strengthening these connections, the approach may enhance both the resilience and responsiveness of One Health systems.

The governance approach in this model is voluntary, non-hierarchical, and locally anchored. It is designed to support coordination without creating a centralised authority or reducing the autonomy of participating communities and organisations. Local actors remain responsible for defining priorities, selecting interventions, mobilising resources, and implementing One Health activities according to their own risks, capacities, and needs. At the same time, the broader network provides a structured mechanism for exchange, learning, and methodological support. Governance is based on facilitation rather than control. Regular online coordination meetings, a rotating Organising Committee, and, where feasible, a small secretariat provide light coordination and administrative support. Thematic Project Working Groups serve as operational bridges between local practice and wider network learning by supporting situation analysis, strategic planning, evaluation, education, communication, and policy dialogue. Decisions are preferably made by consensus, while parallel approaches may be allowed when full agreement is not possible, reflecting the voluntary and flexible nature of participation. In this model, central or higher-level structures do not function as command points. Their role is to provide technical guidance, shared tools, coordination support, and policy alignment. Ownership, decision-making, financing responsibility, and practical implementation remain primarily at the local or regional level. This approach preserves community autonomy while strengthening transparency, trust, information exchange, preparedness, and coordinated action across environmental, animal, and human health sectors.

Equitable global participation is a central principle of the proposed model. One Health priorities should not be defined only from the perspective of high-resource settings, because risks, capacities, and implementation needs differ substantially across regions. Depending on the local context, communities may face different combinations of zoonotic diseases, vector-borne diseases, food safety challenges, environmental degradation, antimicrobial resistance, limited access to veterinary and public health services, or weak surveillance infrastructure. At the same time, available resources vary widely, including funding, trained personnel, laboratory capacity, digital tools, institutional support, and opportunities for cross-sectoral coordination. For this reason, the model emphasises flexible and locally driven participation, allowing each community or region to define its own priorities and adapt the One Health network to its capacities. Low-resource settings should therefore not be seen only as beneficiaries of global One Health initiatives, but as essential contributors of practical knowledge, early warning information, community-based solutions, and experience in working under resource constraints. Strengthening equitable participation increases the relevance, fairness, and practical value of the network, while helping to avoid a top-down approach in which priorities are set mainly by better-resourced institutions or regions.

Such an arrangement does not assume that One Health activities should be financed primarily through central-level mechanisms. Instead, it emphasises locally driven and locally supported implementation, where communities, civil society organisations, professional groups, and relevant local institutions mobilise resources in line with their own priorities and capacities. Central-level structures may still play an important role by providing guidance, coordination, technical support, and policy alignment; however, long-term sustainability is more likely when financial responsibility and ownership are anchored at the local or regional level. This approach makes One Health implementation more practical, resilient, and consistent with its bottom-up logic.

## 5. Conclusions

This approach demonstrates how bottom-up, community-driven action—when structured and networked through voluntary CSO/NGO coordination—can enhance global One Health efforts. The structured One Health network described here offers a practical and scalable model for linking community-based action with global coordination. Integrated evaluation, as standardised templates and iterative improvement cycles, enables comparable evidence generation across heterogeneous contexts; multisectoral collaboration, as the health, veterinary, environmental, and social sectors are linked through structured working groups. The proposed model is ready for empirical testing and offers a roadmap to accelerate evidence-informed, community-driven One Health interventions worldwide. Future work should focus on empirical testing, refinement of tools, and measurement of impact, with the ultimate goal of creating sustainable, equitable, and science-driven One Health systems globally. Such input will be essential for determining whether and how this model can evolve from a conceptual proposal into a practical and evidence-informed One Health implementation approach. Subject to empirical testing, adoption and refinement of this approach may contribute to resilience, equity, and sustainability, while ultimately supporting the protection of human, animal, and environmental health.

## Data Availability

No new data were created or analyzed in this study. Data sharing is not applicable to this article.

## Abbreviations

The following abbreviations are used in this manuscript:

CSO	Civil Society Organisation
NGO	Non-governmental Organisation
SMART	Specific, Measurable, Achievable, Relevant, Time-bound
UN ECOSOC	United Nations Economic and Social Council
PWG	Project Working Group
WASH	Water, sanitation, and hygiene

## Appendix A

### Appendix A.1

**Criteria and Procedures for Identifying and Engaging One Health–Oriented CSOs/NGOs**

This appendix describes the criteria and stepwise procedure for identifying, screening, and engaging civil society organisations (CSOs) and non-governmental organisations (NGOs) to participate in the proposed global One Health coordination network.

*Identification of organisations*

Potential participating organisations are identified through publicly accessible sources, including: One Health-oriented organisational directories and networks; the United Nations ECOSOC civil society registry; and references in peer-reviewed One Health publications and reports.

*Inclusion criteria*

Organisations are eligible for inclusion if they meet all of the following criteria: (i) Explicit engagement with One Health, Planetary Health, or closely related interdisciplinary frameworks; (ii) Legal status as a CSO or NGO (non-profit, non-governmental); (iii) Independence from direct control by governments or commercial entities; (iv) Activity beyond a single disease, species, or narrowly defined intervention; (v) Engagement at national, regional, or international level.

*Exclusion criteria*

Organisations are excluded if they: (i) Are primarily commercial or industry-funded; (ii) Operate exclusively as advocacy groups without programmatic or research activities; (iii) Focus solely on a single pathogen, species, or sector without cross-sectoral integration.

These exclusion criteria apply to eligibility for inclusion as core participating organisations in the proposed coordination network and are not intended to exclude specialist organisations from contributing technical expertise. Organisations with a focused mandate in areas such as, e.g., rabies, antimicrobial resistance, wildlife diseases, vector-borne infections, food safety, or environmental surveillance, may be invited to participate as technical partners, invited experts, members of specific Project Working Groups, or non-voting collaborators where their expertise is relevant. Similarly, commercial or industry-supported organisations with relevant technical knowledge may contribute to clearly defined roles, provided that their participation is governed by transparent conflict-of-interest declaration and management procedures. Such participation should support technical learning and implementation without determining network priorities, governance decisions, or shared outputs.

*Engagement procedure*

Eligible organisations are contacted using a standardised invitation letter (see Appendix A.2). Participation is voluntary. Non-responding organisations may be replaced iteratively to maintain feasibility while preserving transparency.

*Ethical considerations*

Only publicly available organisational information is used. No personal data is collected.

### Appendix A.2

**Invitation Letter and Organisational Information Template**

A. Standardised invitation letter (template)

Subject: Invitation to participate in a global One Health civil society coordination network

Dear [Organisation name],

We invite your organisation to participate in a voluntary global coordination initiative aimed at strengthening community-based One Health action through structured exchange, evaluation, and joint learning among civil society organisations.

Participation does not entail formal membership, financial obligations, or loss of organisational autonomy. The initiative focuses on transparency, collaboration, and shared methodological development.

If you are interested, we kindly ask you to complete the brief organisational information template below.

Sincere,

[Network coordination team]

B. Organisational information template

Participating organisations are invited to provide the following information: (i) Organisation name; (ii) Country or region of registration; (iii) Geographic scope of activities; (iv) Core thematic areas related to One Health; (v) Governance structure (brief description); (vi) Key cooperation partners (optional); (vii) Selected recent publications or outputs (optional).

The purpose of this information is mutual visibility and facilitation of cooperation, not accreditation or ranking.

### Appendix A.3

**Governance Structure, Coordination Mechanisms, and Working Procedures**

The network is governed by the following principles: (i) Voluntary participation; (ii) Transparency; (iii) non-hierarchical coordination; (iv) Respect for organisational autonomy; (v) Inclusiveness and equity.

Coordination mechanisms include: (i) Coordination meetings (Online meetings at least twice per year); (ii) Organising Committee (Small facilitative body with rotating membership); (iii) Secretariat, Optional (Minimal administrative support if feasible); (iv) Project Working Groups (PWGs) are time-bound, thematic groups responsible for specific outputs. Each PWG (i) has a defined mandate and timeline; (ii) is open to interested organisations and experts; (iii) reports progress during coordination meetings.

Decisions are made by consensus where possible. Where consensus cannot be achieved, parallel approaches may be pursued, reflecting the voluntary nature of participation.

Meeting summaries, methodological tools, and agreed outputs are shared openly within the network and, where appropriate, publicly.

Applications of the network should be accompanied by a proportionate data governance arrangement agreed before data collection begins. Data ownership should remain with the organisation, community, institution, or authority that generates or lawfully holds the original data, while the network or relevant Project Working Group may act only as a temporary custodian for agreed purposes of synthesis, learning, and dissemination. Access to shared data should be role-based and limited to authorised contributors, with different levels of access for identifiable, sensitive, aggregated, and public outputs. Confidential or sensitive information, including personal, location-specific, commercially sensitive, or politically sensitive data, should be anonymised, aggregated, or otherwise protected before wider sharing. Cross-border data exchange should occur only where consistent with applicable national laws, institutional requirements, ethical approvals, and agreed data-sharing arrangements. Participating organisations should retain the right to withdraw from future activities; in such cases, procedures should define whether previously contributed anonymised or aggregated data may remain in joint analyses. Local contributors should be appropriately acknowledged, and authorship should follow recognised publication standards, reflecting substantial intellectual, methodological, or field contributions. Empirical projects involving human participants, animal data, identifiable information, environmental sampling, or community-level surveillance should obtain ethical or institutional approval where required. Potential conflicts of interest, including funding, institutional, commercial, or advocacy interests, should be declared and managed transparently. These principles are intended as a concise governance orientation, not as a complete legal framework.

### Appendix A.4

**Community Intervention Fields and SMART-Based Planning Matrix**

This appendix presents the initial menu of intervention fields available to participating communities and the SMART framework used to structure planning and evaluation.

Communities may select one or more of the following seven fields: (i) Surveillance and early warning (Community-based monitoring of health and environmental risks); (ii) Animal health and welfare (Interventions addressing livestock, wildlife, or companion animal health); (iii) Water, sanitation, and hygiene (WASH, Safe water access, sanitation systems, and hygiene promotion); (iv) Food safety and food system (Local food production, processing, and consumption practices); (v) Community awareness and education (Public engagement, risk communication, and behavioural change); (vi) Environmental risks and ecosystem health (Pollution, biodiversity loss, waste management, and ecosystem restoration); (vii) Multisectoral coordination and governance (Local coordination across health, veterinary, environmental, and social sectors).

SMART planning matrix (generic template), Element and Description: (i) Specific (Clearly defined objective and target population); (ii) Measurable (Feasible indicators and data sources); (iii) Achievable (Alignment with local capacity and resources); (iv) Relevant (Relevance to local One Health priorities); (v) Time-bound (Defined implementation and review period).

Communities may adapt indicators and targets to local contexts while maintaining this structure.

To illustrate how the generic SMART-based planning matrix could be operationalised, an example pilot scenario is provided for antimicrobial resistance surveillance in a local One Health setting. This example is not presented as empirical evidence, but as a practical template showing the types of objectives, data fields, indicators, and anticipated evaluation outcomes that participating communities could adapt to their own context (Table A1).

**Table A1:** Illustrative SMART-based planning matrix for a local One Health AMR pilot scenario.

Matrix Element	Example for AMR Pilot Scenario
One Health problem	Antimicrobial resistance risks linked to antimicrobial use and resistant bacteria across human health care, livestock production, food handling, wastewater, and the local environment.
Local setting	Municipality or district with primary health care services, veterinary practices/laboratory, livestock farms, food markets, slaughterhouses or meat processors, wastewater discharge points, and relevant community organisations.
Specific objective	To establish a basic cross-sectoral AMR monitoring and response pilot that connects human, animal, food, and environmental information over a defined period.
Target population/units	Primary health care facilities, veterinary services, livestock farms, food handlers, local market actors, wastewater or surface water sampling points, and community representatives.
Key partners	Local public health institute, veterinary service, municipality, livestock producers, food safety inspectors, laboratories, schools or universities, environmental agency, CSOs/NGOs, and community representatives.
Core data fields	Number and type of samples collected; source of samples; bacterial species or indicator organisms tested; resistance profiles; antimicrobial use practices; hygiene and biosecurity practices; location; date; sector; response actions taken.
Feasible indicators	Number of participating sectors; number of samples collected by sector; proportion of samples with resistant bacteria; number of farms or facilities reporting antimicrobial use practices; number of joint review meetings; number of agreed local prevention measures.
Data sources	Laboratory reports, veterinary records, public health records, farm questionnaires, food safety inspection reports, environmental sampling records, community observations, and meeting summaries.
Achievable activities	Baseline mapping of AMR-related risks; agreement on minimal data fields; limited sentinel sampling; joint interpretation of findings; community education on prudent antimicrobial use, hygiene, food handling, and biosecurity; development of locally feasible prevention measures.
Relevant One Health links	Connects antimicrobial use, human health, animal health, food safety, environmental contamination, community behaviour, and local governance.
Time-bound plan	Six- to twelve-month pilot with baseline assessment, mid-term review, final evaluation, and feedback meeting with local stakeholders.
Anticipated outputs	Local AMR risk profile; shared minimal dataset; summary of cross-sectoral findings; agreed prevention priorities; educational materials; recommendations for strengthening surveillance and stewardship.
Evaluation outcomes	Feasibility of cross-sector data sharing; participation across sectors; completeness of data fields; usefulness of indicators; changes in awareness or practices; identification of barriers to sustain AMR monitoring.
Feedback and learning	Findings are reviewed by local partners and relevant PWGs to refine indicators, improve data collection, compare experience with other sites, and inform future research or policy dialogue.

Note: The indicator proportion of samples with resistant bacteria is intended as an illustrative feasibility indicator for local AMR monitoring. Cross-sectoral and cross-site comparisons would require harmonised epidemiological and laboratory protocols, including sampling design, specimen and bacterial species definitions, bacterial identification methods, antimicrobial susceptibility testing standards, clinical or epidemiological breakpoints, quality-control procedures, isolate deduplication rules, denominators, and minimum metadata.
